# The roles of WRN and BLM RecQ helicases in the Alternative Lengthening of Telomeres

**DOI:** 10.1093/nar/gks862

**Published:** 2012-09-18

**Authors:** Aaron Mendez-Bermudez, Alberto Hidalgo-Bravo, Victoria E. Cotton, Athanasia Gravani, Jennie N. Jeyapalan, Nicola J. Royle

**Affiliations:** Department of Genetics, University of Leicester, University Road, Leicester LE1 7RH, UK

## Abstract

Approximately 10% of all cancers, but a higher proportion of sarcomas, use the recombination-based alternative lengthening of telomeres (ALT) to maintain telomeres. Two RecQ helicase genes, *BLM* and *WRN*, play important roles in homologous recombination repair and they have been implicated in telomeric recombination activity, but their precise roles in ALT are unclear. Using analysis of sequence variation present in human telomeres, we found that a WRN– ALT+ cell line lacks the class of complex telomere mutations attributed to inter-telomeric recombination in other ALT+ cell lines. This suggests that WRN facilitates inter-telomeric recombination when there are sequence differences between the donor and recipient molecules or that sister-telomere interactions are suppressed in the presence of WRN and this promotes inter-telomeric recombination. Depleting BLM in the WRN– ALT+ cell line increased the mutation frequency at telomeres and at the MS32 minisatellite, which is a marker of ALT. The absence of complex telomere mutations persisted in BLM-depleted clones, and there was a clear increase in sequence homogenization across the telomere and MS32 repeat arrays. These data indicate that BLM suppresses unequal sister chromatid interactions that result in excessive homogenization at MS32 and at telomeres in ALT+ cells.

## INTRODUCTION

*BLM*, *WRN* and other members of the RecQ helicase gene family play important roles in many aspects of DNA metabolism, in particular, during replication and repair of DNA double-strand breaks (DSBs) (1,2). DSB repair by homologous recombination (HR) is essential to maintain genome integrity but it requires tight regulation to prevent the accumulation of deleterious effects, such as deletions, duplications, translocations, inversions and loss of heterozygosity. Both Bloom (BS) and Werner (WS) syndromes are autosomal recessive disorders that result in increased genome instability with predisposition to many cancer types in BS and to sarcomas in WS. In addition, both syndromes show early onset of different aspects of normal human ageing and so are regarded as segmental progerias (3).

BLM and WRN have 3′ to 5′ helicase activity and they unwind duplex DNA and promote end resection. *In vitro* BLM and WRN are able to unwind structures that resemble HR intermediates (D-loops and Holliday junctions) and G-quadruplex structures (4,5). They also support branch migration of Holliday junctions over many kilobases (6). The variety of activities supported by RecQ helicases on different DNA structures may make them important players in the selection of the pathway used for DSB repair, with the main outcome being suppression of Holliday junction resolution as crossovers [reviewed in (1,3)]. This suppression also avoids excessive sister chromatid exchange (SCE) throughout the genome, which is a feature of BS [review in (7)] but there is no effect on the genome-wide frequency of SCE in WS (8).

Telomeres pose particular problems during replication because of the well characterized ‘end replication’ problem and the end-processing that is required to generate the single-strand overhangs. In addition, replication forks slow as they encounter the (TTAGGG)_n_ repeat array (9,10), which may be due to the presence of a t-loop or G-quadruplexes that must be removed for the replication fork to pass through the telomeric DNA (11). WRN is required for efficient lagging strand synthesis of telomeres, as deficiency in the mouse results in stochastic sister-telomere loss that can be overcome by expression of telomerase (12,13). It is likely that WRN unwinds G-quadruplex structures during telomere replication and that it plays a role in the formation and resolution of t-loops (14–16). In mouse cells, WRN plays a role in the suppression of post-replication exchange at telomeres [also referred to as telomere-SCE (T-SCE)] (15) and this is also true in normal human cells that lack telomerase, but WRN does not affect SCE formation across the rest of the genome (8,15). The role of BLM at telomeres is less well defined though it also suppresses T-SCE in normal human cells (8), it binds telomeric DNA (17) and it can unwind similar DNA substrates to WRN. In addition, telomeric proteins POT1, TRF1 and TRF2 stimulate the unwinding activity of both WRN and BLM helicases strengthening the link between RecQ helicases and telomere biology (18,19).

A proportion of all tumours, in particular sarcomas, utilize a recombination-based mechanism known as alternative lengthening of telomeres (ALT) to maintain telomeres (20,^21^). The evidence for recombination between non-homologous telomeres in human ALT+ cells comes from plasmid tagging experiments and from mutation analysis of telomeric DNA (22,23). ALT+ cells exhibit an increased frequency of T-SCE, which is another indicator of aberrant recombination activity at telomeres (24,25). Simple exchange between sister-telomeres or between telomeres on non-homologous chromosomes would not maintain telomere length on their own and so the ALT mechanism may involve copying of telomeric sequences from a donor molecule. As a consequence of the aberrant recombination-based activity at telomeres, cell lines and tumours that utilize ALT tend to exhibit highly heterogeneous telomere length (21,26) and they contain extra-chromosomal telomeric DNA in a variety of short linear and circular forms. These extra-chromosomal forms are by-products of uncontrolled telomeric recombination but some may also serve as templates for telomere elongation (27,28). Unexpectedly, a minisatellite (MS32, D1S8 (29)) that comprises a 29 bp GC-rich repeat unit shows extraordinary instability in many ALT+ cell lines and tumours, whereas other GC-rich minisatellites remain stable (30,^31^). The features that distinguish MS32 from other minisatellites in ALT+ cells are not understood, but MS32 instability can be used as a tool to investigate the ALT mechanism.

Evidence that a RecQ helicase plays an essential role in recombination-based telomere maintenance was first shown in *Saccharomyces cerevisiae*, as the yeast RecQ helicase (S*GS1*) is required for ALT-type survivors (32,33). In human ALT+ cell lines, WRN and BLM helicases localize to ALT-associated promyelocytic leukaemia (PML) bodies (27,34,35) and they bind to telomeric DNA (14,17,36) suggesting they both participate in the ALT mechanism. The picture is, however, confusing as immortal human and mouse cell lines that lack telomerase and a functional *WRN* gene have been identified (15,37,38). This indicates either that WRN is not required for ALT or perhaps that these cell lines (WRN– and ALT+) use a different repertoire of recombination-based processes to maintain telomere length. The role of BLM in ALT is also not fully understood, but its interaction with topoisomerase IIIα (TOPO IIIα) (39,40) and other partners are important. Depletion of TOPO IIIα from ALT+ cells reduced the level of TRF2 and BLM. This was accompanied by an increase in telomere-induced DNA damage foci, anaphase bridges and a reduction in cell survival (41). HSP90 and TOPOIIα also interact with BLM, TRF1 and TRF2 specifically in ALT+ cells (42). The specific interactions between BLM and a variety of different proteins support the idea that BLM may play diverse roles at telomeres in ALT+ cells.

To elucidate the roles of *WRN* and *BLM* in the ALT mechanism, we have investigated telomeric DNA sequences in an ALT+ cell line that lacks WRN helicase and then upon depletion of BLM. We present evidence that suggests WRN is required for the formation of complex telomere mutations and we show that BLM suppresses processes that homogenize repeat arrays at the MS32 minisatellite and at telomeres in the WRN– ALT+ cells. A model that is consistent with the data presented and with the known functions of BLM is discussed.

## MATERIALS AND METHODS

### Cell culture

The W-V cell line was donated by Dr Roger Reddel (Children’s Medical Research Unit Sydney, Australia). W-V was derived from a normal fibroblast cell line from a 45-year-old male patient with Werner’s syndrome that was transformed by SV40 infection (43,44). The W-V cell line that survived crisis is a transformed and fully immortalized cell line that does not express telomerase. U2OS cells were a gift from Professor Paolo Salomoni (30). Both cell lines were cultured in Dubbecco’s modified Eagle medium (DMEM) (Gibco, Invitrogen) supplemented with 10% foetal calf serum (FCS; PAA Laboratories). The SUSM-1 cell line was a gift from Professor Olivia M. Pereira-Smith and it was cultured in minimal essential medium (MEM) supplemented with non-essential amino acids and 10% FCS. The Bloom syndrome lymphoblastoid cell line (GM03403, Coriell Cell Repository) was culture in RPMI 1640 with 10% FCS. GM03403 was generated by Epstein-Barr virus infection of B lymphocytes (as for all lymphoblastoid cell lines) from a 33-year-old male donor with Bloom syndrome.

### Single molecule STELA

Allele-specific single molecule (sm)-STELA was used to amplify single telomeres at Xp/Yp or 12q using the XpYp-415C primer or the 12qSTELA primer (5′-CGAAGCAGCATTCTTCTCAG-3′) (31,45,46). sm-STELA was carried out as follows: EcoRI-digested DNA was diluted to 10 ng/μl. A linker oligonucleotide (telorette 2) was added to the diluted DNA to a final concentration of 0.9 μM. The DNA was further diluted with dH_2_O containing tRNA (final concentration 1 ng/μl) so that on an average, each polymerase chain reaction (PCR) produced a single amplicon from one telomere molecule (50–80 pg/PCR). Each STELA comprised a 20 μl reaction containing 1× PCR buffer, 0.5 μM of the allele specific and teltail primer and 1 unit of a mixture of Taq (KapaBiosystems) and Pwo (Roche) polymerase (10:1). The amplification was performed as follows: 25 cycles at 96°C for 20 s, 67°C for 30 s and 68°C for 10 min. STELA products were size separated in a 0.8% agarose gel, transferred to a nylon membrane and hybridized to a ^32^P-labelled telomere probe. Labelled products were detected using a PhosphorImager (Typhoon 9400, GE Healthcare) and analysed using the Image Quant v.2005 software.

### Detection of telomere mutations

The detection of Xp/Yp or 12q telomere mutations was performed using sm-STELA products as templates for Telomere Variant Repeat mapping by PCR (TVR-PCR) to determine the distribution of TTAGGG and sequence-variant repeats (23,45,47). The telomere maps generated were screened for changes that indicated a mutation had occurred. The amplification in TVR-PCR was performed using an allele-specific primer TS-30T at the Xp/Yp telomere or 12q-197A at the 12q telomere, together with a TVR-PCR primer, Tag-TelW, Tag-TelX or Tag-TelY that anneal to TTAGGG, TGAGGG or TCAGGG repeats, respectively.

### Stable short hairpin RNA transfection

To obtain down-regulation of BLM over a longer time period, a pSuperior.neo.gfp plasmid (OligoEngine) containing a DNA sequence for producing a short hairpin RNA (shRNA) against human BLM was constructed (called shBLM). The DNA oligo cloned into the plasmid was 3′**UCACAAGGAAUGAGAAAUA**UUCAAGAGA**UAUUUCUCAUUCCUUGUGA**UU5′, and the shRNA sequence is shown in bold (target is in exon 17). The shBLM (3 μg) was linearized and transfected into (5 × 10^5^) cells using electroporation (Bio-Rad, Gene Pulser XcellTM). The media was supplemented with Geneticin (G418, Invitrogen) at a final concentration of 500 mg/ml 48 h after transfection. Selected colonies were grown for approximately 20 population doublings (PDs) before DNA and proteins were extracted for analysis. Control clones were generated by transfecting the cell lines with linearized pSuperior.neo.gfp vector (empty vector) (46).

### Western blotting and antibodies

Cells (∼1 × 10^6^) were lysed in sodium dodecyl sulphate (SDS)-lysis buffer (150 mM NaCl, 50 mM Tris pH 7.6, 1 mM ethylenediaminetetraacetic acid, 1% Triton) supplemented with 1× protease inhibitor cocktail (Sigma), 2 mM sodium orthovanadate and 2.5 mM sodium fluoride for 20 min at 4°C. Total protein extracts (50 mg) were resolved in 6% SDS-polyacrylamide gel electrophoresis for 1 h at 150 V and transferred to a nitrocellulose membrane. The membrane was incubated for at least 2 h with an antibody against BLM (rabbit 1:2000, AbCam) or 1 h for the loading control β-Actin antibody (mouse 1:12 000, AbCam). Horseradish peroxidise-conjugated secondary antibodies (1:20 000, GE Healthcare) were used to detect primary antibodies. Bound antibodies were detected using the enhanced chemiluminescence (ECL) plus western blotting detection system (GE Healthcare, UK). The membrane was exposed to X-ray film and images analysed using the ImageJ software.

### MS32 minisatellite amplification and mutation frequency analysis

Small-pool PCR was used to amplify the MS32 minisatellite using primers MS32B and MS32E (48). To calculate the mutation frequency, MS32 amplicons that showed a length change (mutation) compared with the common MS32 progenitor alleles were counted across ∼40 small-pool PCR reactions. When more than three mutant molecules appeared to have the same length, it was assumed they had originated from a single event earlier in the expansion of the cell line and so scored as one mutation. To estimate the total number of MS32 molecules screened, the number of amplifiable MS32 molecules was determined using Poisson analysis as described previously (30,^31^).

### Minisatellite variant repeat**-PCR mapping**

The interspersion of sequence variant repeats across the MS32 repeat array was determined by minisatellite variant repeat mapping by PCR (MVR-PCR), as described previously (49). In brief, a flanking primer (MS32O or MS32E) that anneals adjacent to one end of the repeat array was used together with one of four sequence-variant MS32 repeat primers. The reactions were cycled 5× (96°C for 20 s, 65°C for 30 s and 70°C for 3 min) and 13× (96°C 20 s, 63°C 30 s and 70°C for 3 min). The MVR-PCR products were resolved in 1% agarose gels and detected with a ^32^P-labelled MS32 DNA probe (30). The DNA used as template in the MVR-PCR was either a mutant or a progenitor molecule amplified previously by small-pool PCR.

### PCR amplification of MS1 and B6.7 minisatellite

Small-pool PCR was used to amplify MS1 and B6.7 minisatellite using 0.2 μM of primers MS1-420 with MS1+280 (50) and primers 67A with 67B (51), respectively. MS1 amplification was performed as follows: 1 min at 96°C followed by 25 cycles at 96°C for 20 s, 63°C for 30 s and 70°C for 3 min. B6.7 amplification was carried out using 1 min at 96°C followed by 23 cycles at 96°C for 20 s, 60°C for 30 s and 68°C for 5 min. PCR reactions (15–20) were carried out per clonal DNA sample, each reaction contained 100 pg of MboI-digested DNA. The PCR products were resolved in 0.8% agarose gels, transfered to a nylon membrane and detected with a ministatellite-specific probe.

## RESULTS

### Detection of telomere mutations in a WRN negative ALT+ cell line

**Figure 1.**
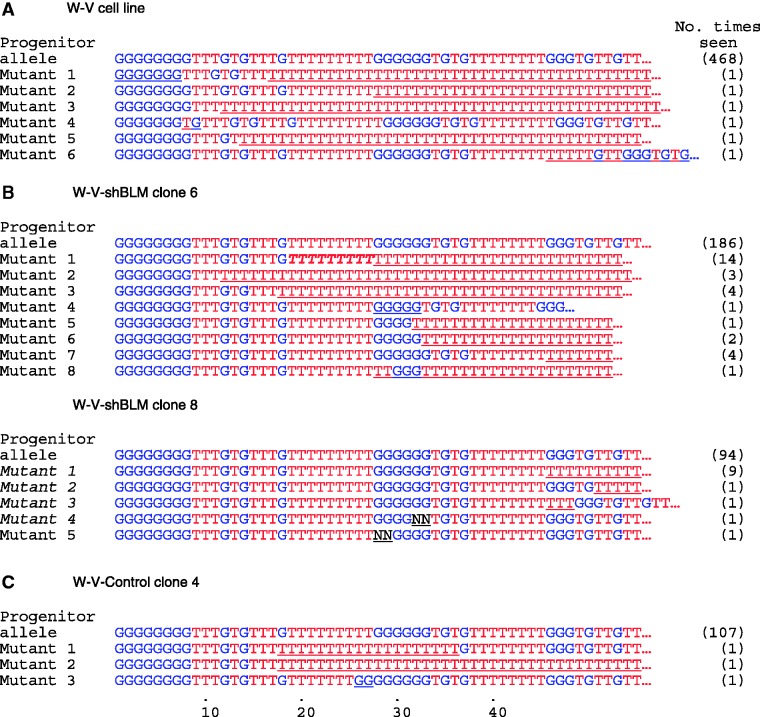
Characterization of Xp/Yp telomere mutations in the W-V cell line and clones transfected with the shBLM or control plasmid. To identify mutations that occur at a low frequency in a population of cells, single telomere molecules that had been amplified by STELA were analysed by TVR-PCR from (**A**) the W-V cell line, (**B**) in two clones expressing the shBLM plasmid and (**C**) in a W-V control clone transfected with the empty vector. The interspersion of TTAGGG repeats (T) and the sequence-variant TGAGGG repeats (G) are shown for the progenitor allele and the mutant molecules. The region(s) where the mutant molecule differs from the progenitor allele is underlined. The number of times each telomere map was observed among the molecules analysed is shown in brackets. The TVR maps for some of the W-V-shBLM clone 8 mutant molecules (in italics) are shown in Supplementary Figure 4. As an example, the block of nine T-type repeats in bold in W-V-shBLM clone 6 mutant 1 identifies the region where the mutation was initiated.

To investigate the role of *WRN* at telomeres in ALT+ cells, we first determined the profile of telomere mutations in an SV40 transformed and immortalized ALT+ cell line derived from a donor with WS (W-V cell line (37,43)). Mutations in telomeres can be identified using TVR-PCR (45,47,52). This allows comparison of the interspersion of TTAGGG and sequence-variant repeats (also referred to as degenerate repeats, see (53)) located within the proximal 1 kb of DNA from the start of the telomere. In order to detect mutations that occur at a low frequency, we amplified single Xp/Yp telomere molecules in the W-V cell line using allele-specific single molecule STELA (sm-STELA) (31,54). The single STELA products were then analysed by TVR-PCR to identify mutations. TVR-PCR utilizes a primer that anneals adjacent to the telomere (chromosome or allele specific) in combination with a primer that binds specifically to TTAGGG or a sequence-variant repeat (e.g. TGAGGG or TCAGGG repeats). We screened 474 Xp/Yp telomere molecules and found six mutations in the W-V cell line (Mutation frequency (6/474), *µ* = 1.3%; Table 1), which is small compared with other ALT+ cell lines (Table 1; (23)). The telomere mutations in the W-V (WRN− ALT+) cell line can most simply be described as follows: four appear as truncations of the interspersion pattern seen in the progenitor allele and replacement with (TTAGGG)*_n_* repeats (mutants 1, 2, 3 and 5; Figure 1A), and one of these mutants was accompanied by loss of one (TGAGGG) repeat; another mutant showed addition of five TTAGGG repeats (mutant 6) and another an addition of one TTAGGG repeat (mutant 4).

**Table 1.** Telomere mutation frequency in WRN+ and WRN– cell lines that utilize the ALT mechanism

ALT+ cell lines	No. of mutant telomere molecules^a^	No. of telomere molecules analysed	Mutation frequency^c^ %
IIICF/a2 (WRN+)^b^	14 (6)	219	6.4 (2.7)
JFCF6 (WRN+)^b^	4 (4)	104	3.8 (3.8)
WI-38VA13/2R (WRN+)^b^	9 (9)	163	5.5 (5.5)
Total	27 (19)	486	5.5 (3.9)
W-V (WRN–)	6 (0)	474	1.3 (0)

^a^The number of complex telomere mutation events is shown in brackets.

^b^Data obtained from (23).

^c^The frequency of complex telomere mutations is shown in brackets.

Mutations at telomeres in ALT+ cell lines have been described previously (23) with an average mutation frequency of 5.5% (Table 1). In the previous studies, the majority (70%) of the telomere mutations were complex in structure, comprising the truncation of the progenitor telomere and addition of a different interspersion of TTAGGG and sequence-variant repeats. The organization of repeats within the complex mutations shows they arise, via an inter-molecular process between non-homologous telomeres or between a telomere and extra-chromosomal telomeric sequence but not by unequal SCE. However, no complex mutations were identified among the 474 telomere molecules analysed from the W-V cell line, which is significantly different from the 19 complex mutations among 486 molecules in other ALT+ cell lines (Table 1; *P* = 0.000003, Fisher exact test 2-tail).

To determine whether the altered mutation spectrum was specific to absence of WRN in the ALT+ cell line, the telomere mutation frequency was investigated in a WS primary fibroblast cell line that had been immortalized by telomerase expression (AG03141B + hTERT). Three telomeres (associated with XpYp flanking haplotype A; XpYp flanking haplotype B and 12q flanking haplotype B) were analysed in each of 150 clonally derived DNA samples from the AG03141B+hTERT cell line, and no mutations were identified (mutation frequency *µ* = 0 (0/450); Hilda Pickett and N.J.Royle., unpublished data). This mutation frequency is significantly different from that seen in the W-V cell line (*P* = 0.031, Fisher exact test, 2-tail) but in line with other primary cells lines (23). In summary, the data presented show that the W-V cell line, lacking the WRN protein, does not form the complex telomere mutations derived from inter-telomeric processes.

### Down-regulation of BLM in the W-V cell line

To investigate the role of BLM at telomeres, we analysed the telomere mutation frequency and profile in a BLM-deficient lymphoblastoid cell line, GM03403, derived from a donor with Bloom syndrome. The 12q telomere investigated contained an intricate pattern of TTAGGG repeats interspersed with TGAGGG and other repeats [as expected at 12q (47)]. Two mutations were identified at the 12qA1-associated telomere among 131 molecules analysed (*µ* = 1.5%, Supplementary Figure S1). Although this mutation frequency is higher than those measured in normal or telomerase+ cell lines (23), the difference is not statistically significant. Both mutations were small intra-allelic deletion mutations of approximately three repeats. This is consistent with the deletion mutations seen at DSBs in a BLM-depleted SV40 immortalized cell lines (55,56) and in other organisms (57).

**Figure 2.**
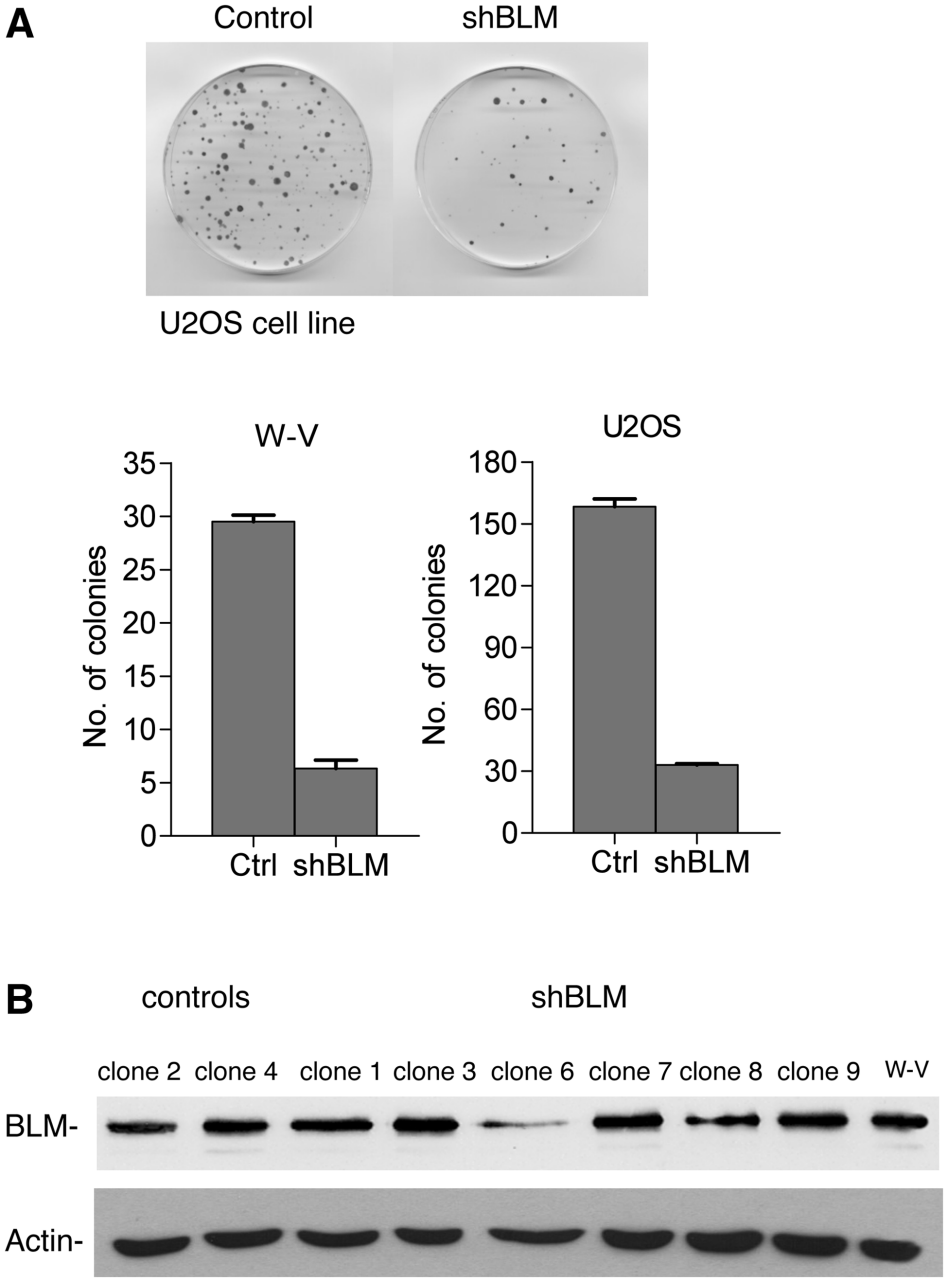
Reduction of BLM expression reduces colony formation in ALT+ cell lines. (**A**) Colony forming assays in the U2OS and W-V ALT+ cell lines. Cells were transfected with the shBLM or empty vector (ctrl) plasmids and after two weeks in selective medium the plates were fixed, stained and colonies counted. The histograms show the results from three transfection experiments, in which 5 × 10^5^ cells were transfected with 3 µg of plasmid. The error bars show standard error of the mean (SEM). (**B**) western blot analysis of clones isolated for further analysis. The W-V parental cell line, two control clones (empty vector) and nine clones transfected with the shBLM-plasmid are shown. Among the shBLM clones, only clones 6 and 8 show reduced BLM expression.

To investigate the role of BLM in the ALT mechanism, we then chose to disrupt BLM expression in the W-V cell line, which already lacks the WRN protein. A vector encoding a short hairpin RNA against BLM was constructed, and the effect of BLM down-regulation on colony formation was investigated in two ALT+ cell lines, W-V (WRN–) and U2OS (WRN+; Figure 2A). The number of colonies formed in the W-V and U2OS cell lines transfected with the shBLM was only ∼20% of that seen in cells transfected with an empty vector control. This significant reduction in colony forming efficiency (Student’s *t*-test *P* < 0.0001 and *P* = 0.0011, respectively) indicates that BLM is important for cell growth in ALT+ cells. This has been reported previously by Temine-Smaali *et al.* who showed that RNA interference-mediated depletion of BLM reduced the growth rate of U2OS (ALT+) cells but did not have a significant effect on HeLa (telomerase+, tumour-derived cell line) (41). Others have shown that the colony forming efficiency is reduced in a BLM-deficient HeLa cell line (58) but to a lesser extent than seen here in ALT+ cells lines, suggesting that BLM depletion may have a greater effect in ALT+ cell lines.

Next, the shBLM plasmid or empty vector control was transfected into W-V cells and clones were isolated and expanded. After 20–21 PDs, the level of BLM expression was determined by western blot analysis. Most of the isolated clones showed very little or no BLM down-regulation. However, one clone (W-V-shBLM clone 6) showed a reduction of ∼60% (Figure 2B) and two other clones (W-V-shBLM clones 2 and 8) showed a reduction of ∼20% when measured at PD20.

### BLM down-regulation increases MS32 minisatellite instability

As stated earlier in the text, ALT+ cell lines and tumours are characterized by extraordinary instability at the MS32 minisatellite (D1S8) on chromosome 1 (30,^31^). This minisatellite is composed of a 29 bp GC-rich repeat array that is highly unstable in the germ-line but not in normal somatic cells (59,60). The meiotic cross-over and conversion processes that underlie germ-line instability at this locus seem to be driven by a recombination hotspot adjacent to the repeat array. In ALT+ cell lines, the MS32 mutation rate varies but is on an average 55 times higher than in ALT− cell lines, whereas six other minisatellites remain stable ((30) and unpublished data).

**Figure 3.**
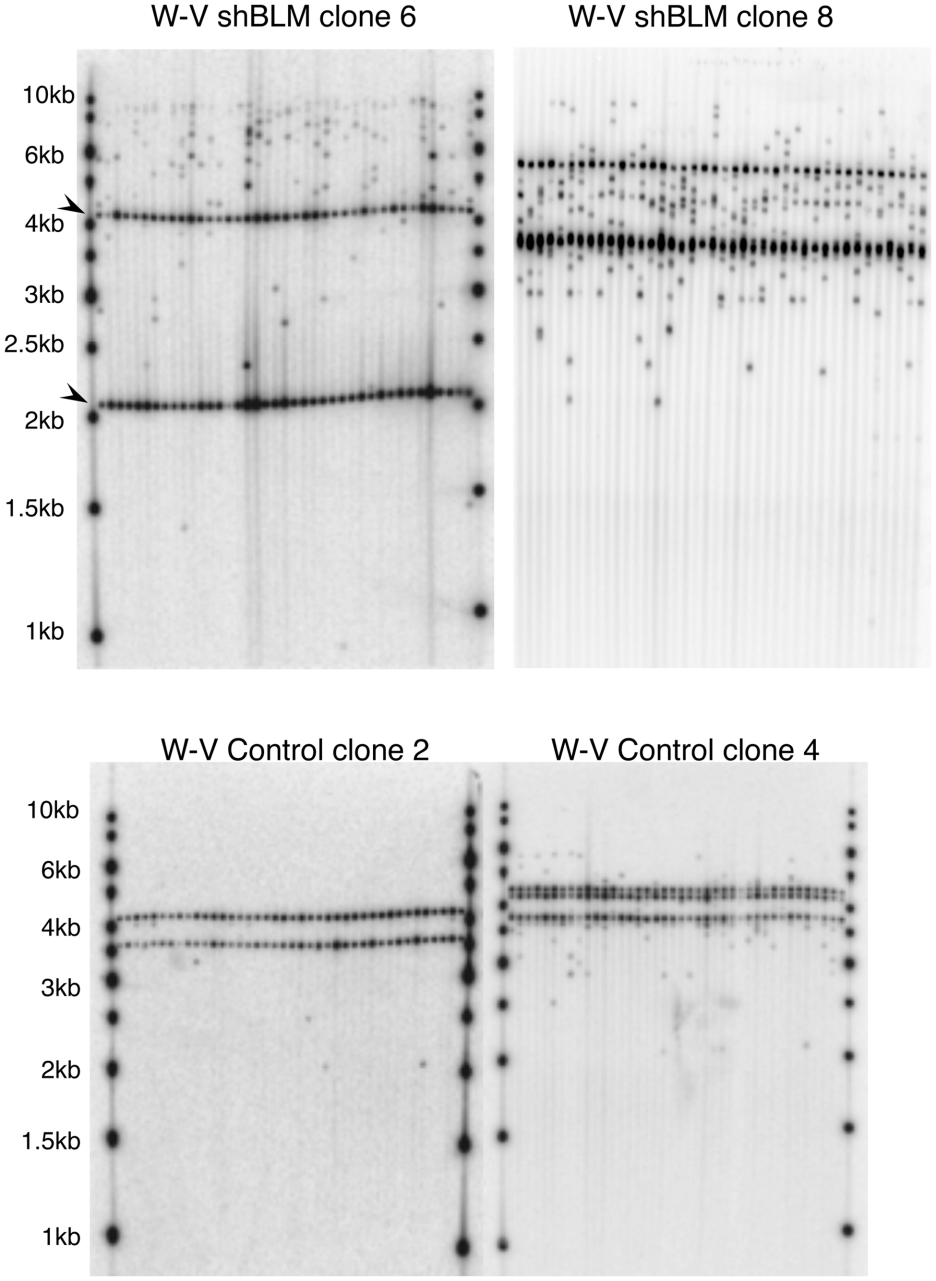
Small-pool PCR detection of MS32 length mutant molecules in shBLM-plasmid and control clones. The tracks show the results of MS32 amplification from small aliquots of DNA followed by detection by Southern blot hybridization. The common or more stable alleles in each clone are present in all tracks across the gel and identified with arrows in W-VshBLM clone 6. Mutant molecules that differ in size from the progenitor bands were counted.

We checked MS32 stability in the BLM-negative GM03403 lymphoblastoid cell line, the mutation frequency was low (1.2 × 10^−^^3^, Supplementary Figure S2) and in line with other telomerase-expressing immortal cell lines (30). Therefore, the absence of BLM alone does not result in high MS32 instability. We then determined the average mutation rate at MS32 in six empty vector control clones derived from the W-V cell line and compared it with the average mutation rate from three clones transfected with the shBLM plasmid, W-V-shBLM clones 6, 2 and 8, which showed varying levels of BLM depletion (Figure 3; Table 2). The MS32 mutation rate increased ∼6-fold when BLM was down-regulated (7.2 × 10^−^^3^ vs 1.1 × 10^−^^3^, *P* = 0.028, Mann–Whitney U test, Table 2). To check whether the increased MS32 instability in W-V-shBLM clones arose from a general increase in genome instability, two other minisatellites, MS1 and B6.7, were investigated in W-V-control clones 2 and 4 and the W-V-shBLM clone 6 (Supplementary Figure S3). Neither minisatellite showed instability. Therefore the data indicate that BLM specifically suppresses MS32 instability in ALT+ cells.

**Table 2.** MS32 mutation rate in control and shBLM clones

Clone (BLM down regulation %)				
	No. Mmutant molecules^a^	Mutation frequency^b^	No. PDs	Mutation rate per PD
W-V-Control clone 2	5	0.0036	20	1.8 × x10^-4^
W-V-Control clone 3	25	0.0191	22	4.6 × x10^-4^
W-V-Control clone 4	56	0.0434	21	7 × x10^-–4^
W-V-Control clone 8	23	0.0481	20	2.4 × x10^-–3^
W-V-Control clone 10	11	0.0184	21	7 × x10^-–4^
W-V-Control clone 11	36	0.0487	20	2.4 × x10^-–3^
Average mutation rate in control clones = 1.1 × x10^-–3^				
W-V-shBLM clone 6 (60%)	103	0.2084	20	1 × x10^-–2^
W-V-shBLM clone 2 (20%)	110	0.0914	20	4.6 × x10^-–3^
W-V-shBLM clone 8 (20%)	160	0.1424	20	7.1 × x10^-–3^
Average mutation rate in shBLM clones = 7.2 × x10^-–3^				

^a^Mutant molecules that differed in length to the ‘progenitor’ alleles (see Figure 3) were counted.

^b^The total number of molecules analysed was estimated (see Materials and Methods section) and used to calculate the mutation frequency and mutation rate per PD (population doubling).

### Analysis of MS32 mutations in BLM depleted W-V clones

In order to understand the role of WRN and BLM in the mutation mechanism that underlies the increased instability at MS32, MVR-PCR was used to characterize MS32 mutations in the W-V cell line and in the W-V-shBLMclone6. In brief, MS32 amplifications from small aliquots of genomic DNA were conducted and single MS32 molecules that showed length mutations were identified. The PCR product of each MS32 mutant molecule was diluted and used as template for MVR-PCR mapping from each end of the repeat array (49).

**Figure 4.**
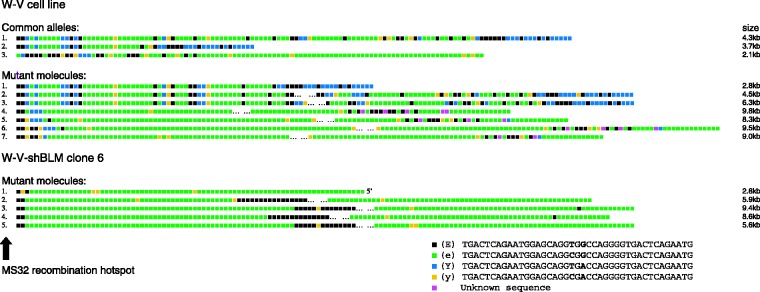
Partial characterization of MS32 mutant molecules in the W-V parental cell line and W-VshBLM clone 6. The order of MS32 sequence-variant repeats was determined by MVR-PCR from the flanking sequence at each end of the repeat array. The complete MVR maps are shown for the three common alleles in the W-V cell line, and comparison suggests that allele 2 was derived from allele 1. MVR maps are shown for seven different length mutant molecules from the W-V cell line and five from the W-V-shBLM clone 6. It was not possible to determine the complete MVR maps for long molecules and so gaps are shown ( … ) to indicate missing data. The four sequence-variant repeats within the MS32 minisatellite are Y, y, e and E (49). The location of the germline recombination hotspot, adjacent to one end of the minisatellite, is indicated with an arrow.

The W-V cell line carries three copies of chromosome 1q in some but not all cells (N.J.Royle, unpublished data), and three fairly stable MS32 bands (common alleles) were detected in the W-V cell line composed of 54, 106 and 126 repeats (Figure 4). MVR maps from length-mutant MS32 molecules from the W-V cell line were compared with the mapped alleles. Most of the mutant molecules were much larger than the common alleles and so partial MVR maps have been produced, as it was not possible to complete the maps across the middle of the large mutant molecules. Three of the mutant molecules obtained from the W-V cell line (mutant molecules 1, 2 and 3) showed similarities to the mapped allele 1 at each end but they differ through a series of deletion and insertion events throughout the array (Figure 4). The relationship between the mapped alleles and mutant molecules 4–7 is not obvious. In addition, these mutants are dominated by expansion of one repeat-type (e-type; Figure 4) showing that the mutation process in the W-V cell line often results in homogenization of the repeat array.

The five mutant MS32 molecules analysed from the W-V-shBLM clone 6 contain expanded tracts of e-type repeats and mutants 2–5 are interrupted by a block of E-type repeats. It is difficult to relate the block-like structure of these mutants directly to the mapped common alleles in the W-V cell line, though allele 3 contains the longest homogenous array of e-type repeats. It seems that following depletion of BLM, the MS32 repeat array has undergone further homogenization and expansion (W-V-shBLM clone 6 mutants 1–5; Figure 4) indicating that BLM suppresses this in ALT+ cells.

The homogenization and expansion of the MS32 repeat arrays in mutants 4–7 from the WRN-deficient W-V cell line and in mutants from the W-V-shBLM clone 6 are likely to be attributable to unequal interactions between sister chromatids. This may include repeated strand invasion and copying between sister chromatids via synthesis-dependent strand annealing (SDSA) or perhaps break-induced replication (BIR) between sister chromatids (7,61,62).

### BLM down-regulation increases telomere instability

There is evidence that BLM plays a role at telomeres in ALT+ cells (35,36,41,42), but what it does remains unclear. We have shown previously that MS32 instability is closely associated with ALT activity (30,^31^) and so the increased MS32 instability in BLM-depleted W-V cells indicated that mutational activity at telomeres may also be affected. To investigate this, allele-specific single molecule, STELA at Xp/Yp followed by TVR-PCR, was used to investigate the telomere mutation rate and profile in W-V clones. In the W-V-shBLM clone 6 (∼60% BLM depletion at the point of assay), 30 mutant molecules were detected among 216 molecules analysed giving a mutation rate of 6.9 × 10^−^^3^ per PD, comparable with the telomere mutation rate obtained in WV-shBLM clone 8 (∼20% BLM depletion at the point of assay; Table 3). Similar analysis was carried out in the W-V-Control clone 4, which showed a lower mutation rate (1.3 × 10^−^^3^ per PD) than that seen in shBLM clones. The effect on telomere mutation rate in W-V-shBLM clone 8 was unexpected, as the clone showed only modest down-regulation (∼20%) when it was measured (following ∼20 PD clonal expansion, Figure 2). Furthermore, the BLM expression level five PDs later in both the W-V-shBLM clone 8 and W-V-shBLM clone 6 had reverted to that seen in controls. Therefore, it seems likely that the level of BLM down-regulation was higher in W-V-shBLM clone 8 immediately following transfection with the shBLM plasmid but it was gradually lost during clonal expansion, although during this time telomere mutations accumulated. This then explains why the telomere and MS32 mutation rates are similar to that seen in the W-V-shBLM clone 6.

**Table 3.** Telomere mutation analysis at the Xp/Yp telomere

Sample	Total no. of mutant molecules^a^	Total no. of (TTAGGG)_n_ homogenising mutations	No. of molecules analysed	Mutation frequency^b^	Mutation rate^c^
W-V cell line	6 (6)	4 (0.84%)	474	0.013 (0.013)	ND
W-V-shBLM clone 6	30 (8)	29 (13.4%)	216	0.138 (0.037)	6.9 ×10^−3^ (1.85 ×10^−3^)
W-V-shBLM clone 8	13 (5)	10 (9.5%)	107	0.121 (0.047)	6.1 ×10^−3^ (2.35 ×10^−3^)
W-V-control clone 4	3 (3)	1 (0.9%)	110	0.027 (0.027)	1.3 ×10^−3^ (1.3 ×10^−3^)

^a^The number of different mutations observed among the total analysed is shown in brackets.

^b^The frequency of different mutations observed is shown in brackets.

^c^The mutation rate per population doubling was calculated for the total number of mutations and in brackets for the number of different mutations. The number of PD is from Table 2. ND, not determined.

Interpretation of the telomere mutation rates based on the total count of mutants detected must be done cautiously because both the W-V-shBLM clone 6 and clone 8 contained some mutant structures that were seen more than once (Figure 1 and Supplementary Figure S4), though this was not seen in the W-V cell line or in the W-V-Control clone 4. We ruled out a problem of contamination since the single molecule STELA products used for TVR-PCR mapping were from different sized amplicons, showing they come from different telomere molecules. The repeated isolation of mutant molecules with the same interspersion pattern could arise from somatic mosaicism, from independent mutation events or a combination. For example, W-V-shBLM clone 6 mutant 1 was detected in 14 times (Figure 1), and so it is not known whether they represent 14 independent mutation events or, at the other extreme, one mutation event that occurred early in the expansion of the clone (somatic mosaicism). The breakpoint in the W-V-shBLM clone 6 mutant 1 maps to a block of nine TTAGGG repeat types (in bold italics in Figure 1) that may be a favoured site, in which case, some of the 14 copies of mutant 1 may have arisen independently. There is evidence to support this because mutant 2 in the W-V cell line (Figure 1) has the same structure but must have arisen independently from mutant 1 in clone 6. Similarly, another mutant that was detected three times, W-V-shBLM clone 6 mutant 2, has the same structure as the W-V mutant 3 and W-V-shBLM clone 6 mutant 3 (detected four times) has the same structure as W-V-Control clone 4 mutant 2.

In summary, the telomere mutation rates in W-V-shBLM clones 6 and 8 cannot be determined exactly, because of the unknown contribution of somatic mosaicism, but the data presented show the telomere mutation rate increased when BLM was down-regulated. In addition, mutations that resulted in homogenization of the telomere repeat array occurred much more often in the BLM-depleted clones (13.4% and 9.5% in W-V-shBLMclone 6 and 8, respectively) than in the control or W-V parental cell line (0.9 and 0.84% in W-V control clone 4 and W-V, respectively; Figure 1; Table 3). In contrast, telomere repeat array homogenization was not evident in the BLM-deficient lymphoblastoid cell line (GM03403; Supplementary Figure S1). Thus BLM depletion in this WRN-ALT+ cell line (W-V) specifically increased the rate of mutation processes that remove variant repeats (as seen at MS32) and so tended to homogenize the repeat array towards pure (TTAGGG)*_n_*.

## DISCUSSION

Cells that utilize the ALT pathway support a variety of recombination-based activities that involve telomeric DNA sequences (27). Evidence for a recombination-based or BIR-like mechanism between non-homologous telomeres or between telomeres and extra-chromosomal telomeric repeats comes, in part, from analysis of the interspersion pattern of sequence-variant repeats present at the start of human telomeres (23,63). Complex telomere mutations, in which the interspersion pattern of the progenitor telomere was truncated and replaced by a different interspersion pattern distal to the fusion point, have been detected in 3.9% of telomeres in ALT+ cells but not in telomerase expressing or normal cell lines and such mutations may arise by BIR. In this study, we have shown that the W-V ALT+ cell line derived from a donor with WS lacks this class of complex telomere mutations (0/474 telomere molecules analysed). This raises the possibility that formation of a complex telomere mutation, which involves strand annealing between two non-identical telomere sequences (with different interspersion patterns), requires the WRN protein, possibly because of its exonuclease activity.

To test this further, we attempted to express WRN in the W-V cell line to determine if this resulted in the formation of complex telomere mutations. We used the pLPC-WRN plasmid (12) and selected 10 clones under puromycin selection, but we were unable to detect WRN expression by western blot analysis after ∼20 PDs of clonal expansion. Therefore, other approaches will be needed to investigate further the role of WRN in the formation of complex telomere mutations in ALT+ cells. Remarkably, another WRN- immortalized cell line (AG11395), which does not express telomerase, uses an alternative route to telomere length maintenance that resembles the yeast Type 1 survivors (38,64,65). This also suggests that WRN plays an important role in the recombination-based mechanism (similar to yeast Type II survivors (64)) used to maintain telomeres in the majority of ALT+ cell lines and tumours.

The absence of complex telomere mutations in the W-V cell line is notable but mutations that arise from intra-allelic processes were identified (Figure 1A). In particular, four of the six characterized mutations showed loss of variant-telomere repeats leading to homogenization of the telomere repeat array. These observations suggest there has been a shift away from inter-telomeric interactions in the WRN deficient W-V cell line (i.e. between telomeric sequences that are not identical) towards interactions between sister chromatids. Therefore, this raises the possibility that the sister-telomere interactions (or their resolution as exchanges) in ALT+ cells are suppressed by WRN, as reported in normal cells (8). BLM is also know to suppress T-SCEs (8), but the BLM protein expression is normal in the W-V cell line (Supplementary Figure S5) suggesting it is not contributing to the altered telomere mutation profile in the W-V cell line. Alternatively, a gene that regulates BLM activity may have altered expression, for example, HSP90 that inhibits BLM helicase activity in ALT+ cells (42)).

Down-regulation of BLM expression in W-V clones increased the telomere mutation rate, primarily through an increase in mutations that homogenize the telomere repeat array (Table 3; Figure 1). Thus, it seems likely that BLM depletion resulted in further deregulation of sister-telomere interactions, leading to strand invasion between misaligned sister-telomeres, with resolution either as deletion events when BLM was depleted from the W-V cells that already lack WRN (55–57,66,67) or to strand invasion between misaligned sister-telomeres and copying from the donor sister-telomere, in a BIR-type process. Further evidence that BLM plays a role in the suppression of telomere homogenising mutations comes from another ALT+ cell line, IIICF/a2. We showed previously that this cell line supports complex telomere mutations and mutations that homogenize the telomere repeat array (23), similar to the mutations characterized here in the W-V cell line. Interestingly, the IIICF/a2 cell line shows reduced BLM expression (approximately 60% reduction in protein level) compared with other ALT+ cell lines (Supplementary Figure S5). In addition, Muntoni *et al.* showed that a related IIICF/c ALT+ cell line supports telomeric intra-allelic DNA copying (68).

Previous studies by Bhattacharyya *et al.* (42) suggested that down-regulation of BLM in the SAOS-2 ALT+ cell line led to a rapid reduction in telomere length and they proposed that this may be due to failure to restart stalled replication forks at telomeres (69). We were unable to measure median telomere length, when BLM expression was down-regulated in W-V-shBLM clone 6 because there was insufficient DNA for Southern blot analysis but telomere length was similar to control clones from PD28 onwards (soon after BLM expression had reverted to the normal level). Further work will be needed to determine whether BLM depletion affects telomere length in ALT+ cell lines.

Instability at MS32 in ALT+ cells shows deletions, insertions and complex shuffling or conversion of repeats suggesting that mutant molecules arise from multiple rounds of strand invasion and repair by SDSA between misaligned sister chromatids (30,^66^,^67^). The MS32 minisatellite is unstable in the WRN negative W-V cell line but interestingly some mutant molecules included expansions of blocks of a single repeat-type and loss of other sequence-variant repeats (Figure 4). The loss of variation in some MS32 mutant molecules is similar to mutations seen at telomeres in the W-V cell line but without evidence of a bias towards deletions. Moreover, BLM down-regulation in the W-V clones increased the MS32 mutation rate (∼6-fold) compared with control clones, without affecting two other minisatellites (Supplementary Figure S3). The mutant MS32 molecules in the BLM-depleted W-V clone (W-VshBLM clone 6; Figure 4) showed further expansion of blocks of a single repeat type with loss of sequence variation from the ends of the repeat arrays. The block-like structure present in some MS32 mutants is reminiscent of the minisatellite on the human Y chromosome (MSY1, (70)) that evolves along haploid lineages, driven by intra-allelic processes that may arise by SDSA between misaligned sister chromatids (71). In summary, the homogenization of the MS32 repeat array in the W-V shBLM clone 6 is similar to the types of mutations seen at the telomeres. These data are another indicator that MS32 instability is tightly linked to the ALT mechanism and that loss of WRN and depletion of BLM have specific effects on ALT.

**Figure 5.**
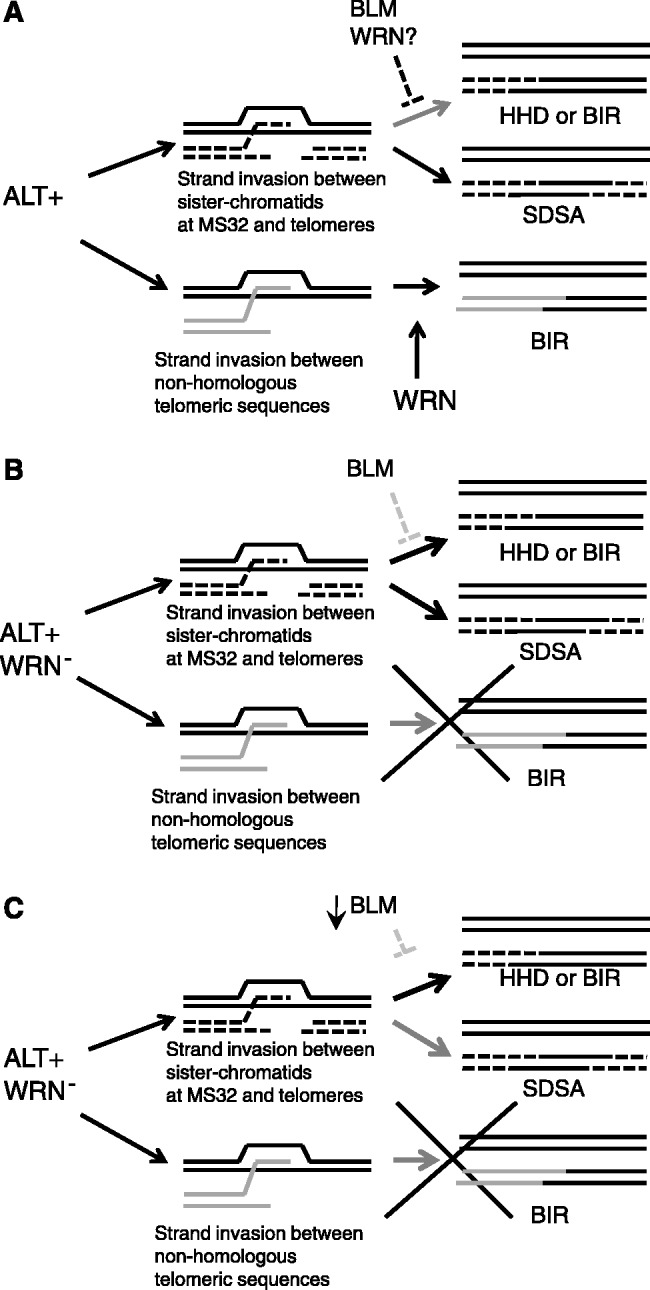
Model for the roles of WRN and BLM at telomeres and MS32 in ALT+ cells. (**A**) In cells that utilize the ALT pathway, telomeres can be extended by strand invasion and copying from a non-homologous telomere by a BIR mechanism that is promoted by WRN (lower arrow). In ALT+ cells, double strand breaks at MS32 (and sometimes telomeres) can be repaired via SDSA, which may involve multiple rounds of strand invasion between misaligned sister chromatids, copying and disassociation (upper arrow). However, BLM and perhaps WRN suppress outcomes that result in repeat sequence homogenization (through homology dependent deletions (HDD) or BIR-type events). (**B**) In the WRN negative ALT+ (W-V) cell line, BIR between non-homologous (non identical) telomeres is reduced or absent (lower arrow). Suppression of double-strand break repair that leads to HDD or BIR-type events between misaligned sister chromatids at MS32 and at telomeres is relaxed, possibly because WRN is absent or suppression by BLM is relaxed, and mutations that result in homogenization of the repeat arrays are seen. (c) Down-regulation of BLM in a WRN negative ALT+ cell line further reduces suppression of the events between sister chromatids at MS32 and at telomeres and the frequency of mutant molecules that show repeat homogenization increases.

In conclusion, we propose a model for the roles of WRN and BLM at telomeres and the MS32 minisatellite in ALT+ cell lines (Figure 5). The absence of complex telomere mutations in the WRN negative ALT+ cell line (W-V) indicates that WRN facilitates the process that underlies these mutation events. WRN may directly promote the formation or the resolution of telomere–telomere interactions when there are differences between the donor and recipient molecules (Figure 5A, lower arrow). WRN may also suppress interactions between sister telomeres (8), and this suppression may increase the likelihood of telomere–telomere interactions. Thus, in the absence of WRN, telomere mutations are driven through intra-allelic processes, including unequal sister-telomere interactions (Figure 5B) that are also suppressed by BLM (8,15,24). Consequently, depletion of BLM further relaxes suppression of the sister chromatid driven mutation events at MS32 and at telomeres so explaining the increase in mutation rates, via an increase in mutations that homogenize the repeat arrays.

## SUPPLEMENTARY DATA

Supplementary Data are available at NAR Online: Supplementary Figures 1–5.

## FUNDING

Cancer Research, UK [C17992/A8641 to N.J.R.]; the Medical Research Council (MRC) [G0500336 to N.J.R.]; Conacyt, Mexico (to A.H.-B.). Funding for open access charge: MRC.

*Conflict of interest statement*. None declared.

## Supplementary Material

Supplementary Data
