# Supplementary material for: The roles of WRN and BLM RecQ helicases in the Alternative Lengthening of Telomeres
Source: Nucleic Acids Res. 2012 Sep 18;40(21):10809–20. doi: 10.1093/nar/gks862 (PMC3510502; doi:10.1093/nar/gks862)
Supplement: Supplementary Data [file supp_40_21_10809__index.html]

The roles of WRN and BLM RecQ helicases in the Alternative Lengthening of Telomeres — The roles of WRN and BLM RecQ helicases in the Alternative Lengthening of Telomeres — Supplementary Data 

# The roles of WRN and BLM RecQ helicases in the Alternative Lengthening of Telomeres

## Supplementary Data

files

**Files in this Data Supplement:**

- Supplementary Data - pdf file
