# Supplementary material for: The roles of WRN and BLM RecQ helicases in the Alternative Lengthening of Telomeres
Source: Nucleic Acids Res. 2012 Sep 18;40(21):10809–20. doi: 10.1093/nar/gks862 (PMC3510502; doi:10.1093/nar/gks862)
Supplement: Supplementary Data [file supp_gks862_nar-00347-f-2012-File007.pdf]

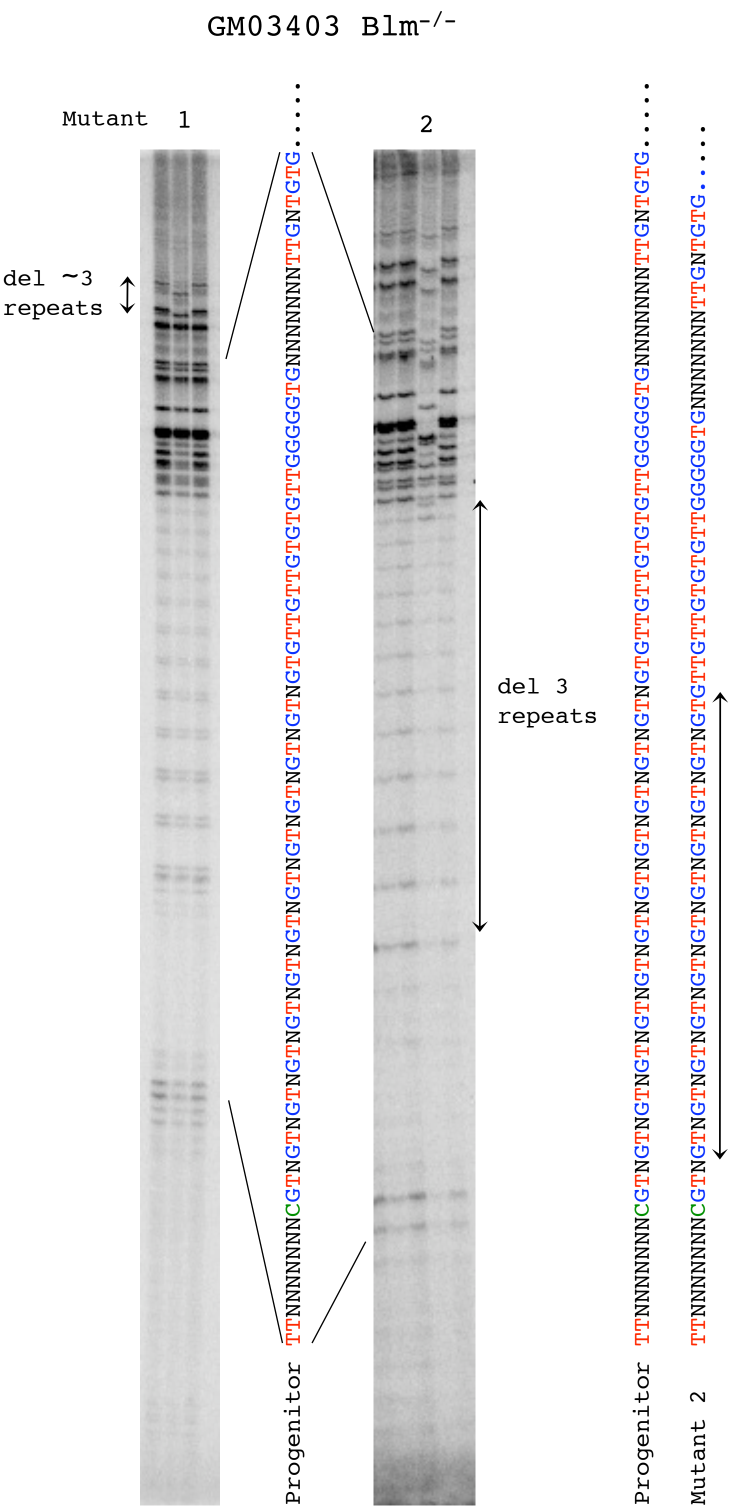

Supplementary Figure 1

Telomere mutation detection in GM03403 (BLM^{-}) lymphoblastoid cell line.

Allele specific STELA was conducted at the 12qA1 associated telomere; amplicons from 131 single molecules were isolated and analysed by TVR-PCR. Two mutant molecules were identified giving a mutation frequency μ= 0.015 (1.5%). Each mutant showed a simple deletion.

Each gel image shows the distribution of TTAGGG repeats in one mutant molecule flanked by non-mutant molecules that have the map of the progenitor allele. The telomere starts with two TTAGGG repeats near the bottom of the gel. The interspersion of TTAGGG and variant repeats in the progenitor allele is shown as a letter code or telomere map (T, TTAGGG; G, TGAGGG; C, TCAGGG; N, unknown repeat) in between the two gel images. Limitation of the gel resolution reduces the accuracy of reading the telomere map at the top of the acrylamide gel, though it is clear that the map continues (.....).

The deletion in mutant 1 is located near the top of the gel (double headed arrow next to the gel) and comprises a simple loss of ~3 repeats. The approximate location of the deletion in mutant 2 is show by the long double-headed arrow next to the gel. It comprises a loss of three repeats (NGT) from within a block of repeats with a higher order structure $(NGT)_{12}$.

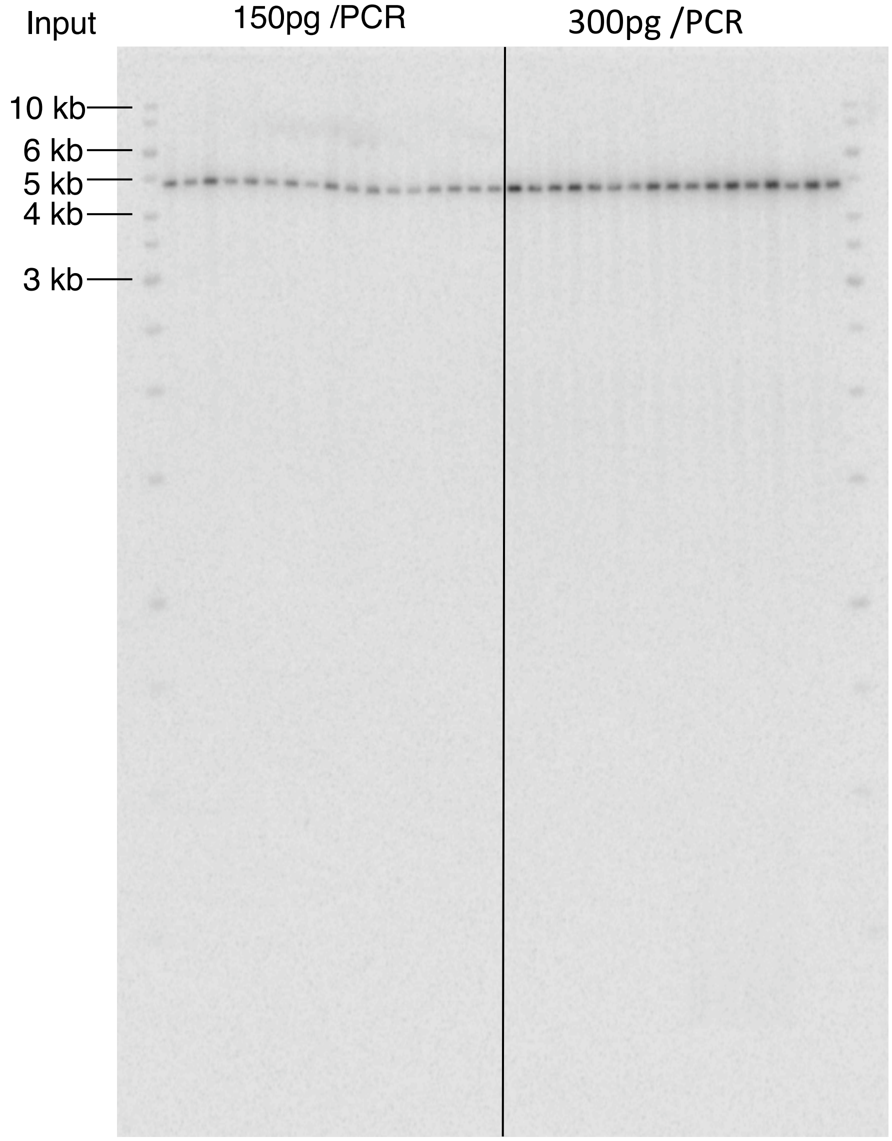

Supplementary Figure 2

The MS32 minisatellite did not show a high level of instability in a lymphoblastoid cell line from a Bloom syndrome patient $BLM^{-/-}$

The phosphor image shows an example of small pool PCR analysis across the MS32 repeat array, conducted on genomic DNA from GM03403, a lymphoblastoid cell line derived from a Bloom syndrome patient. Genomic DNA, ~150pg or ~300pg per PCR, was amplified with primers MS32B and MS32E as described in the materials and methods. The constant band present in all the tracks represents the amplified product from one MS32 allele. It is likely that the second allele is very large and so does not amplify efficiently in the PCR reactions. In summary, three independent MS32 mutant molecules were identified (not shown) among an estimated 2550 molecules screened. The mutation frequency (0.0012 / molecule) is similar to other telomerase positive cells lines but much lower than in ALT+ cell lines.

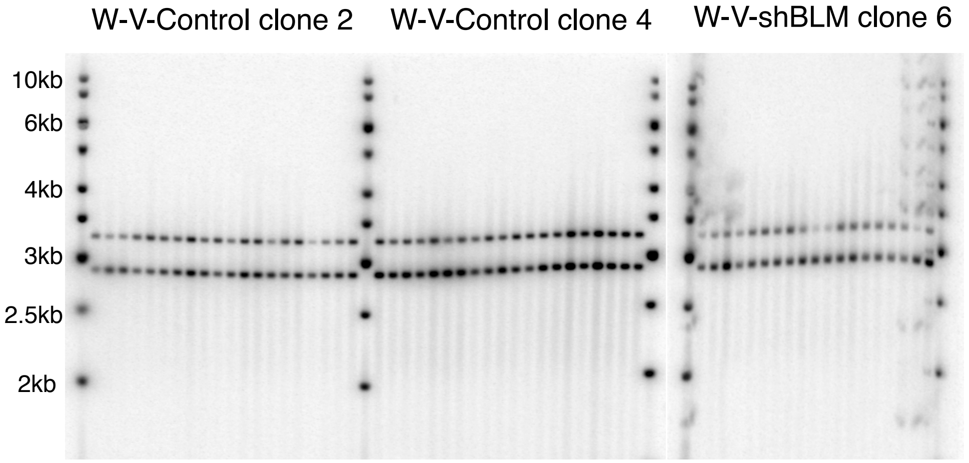

MS1

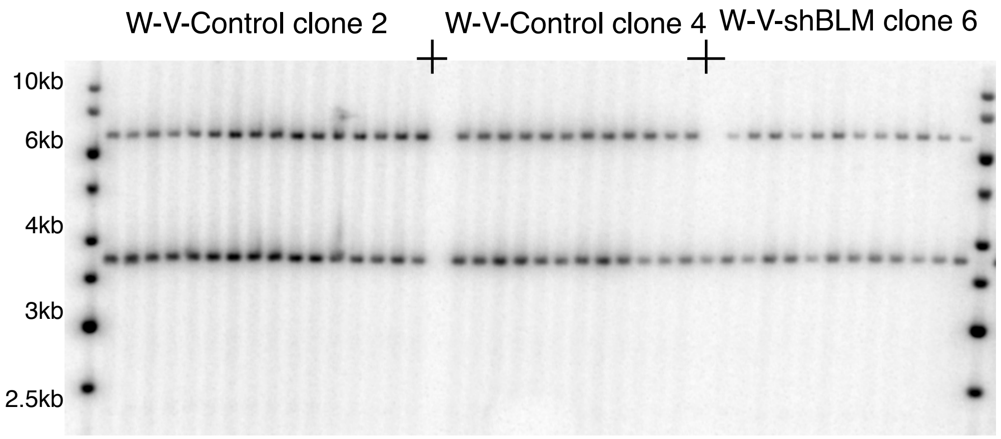

B6.7

Supplementary figure 3.

The MS1 (D1S7) and B6.7 minisatellites remain stable in ALT+ cells that lack Wrn and following down-regulation of Blm.

Small pool PCR across the MS1 and B6.7 repeat arrays was conducted on DNA from W-V shBLM clone 6 and two empty vector control clones. Each small pool PCR (one track in the gel) was conducted on ~100pg of genomic DNA. An estimated 330 MS1 molecules and 250 B6.7 molecules were screened for mutations in each clone. No mutant molecules were observed indicating that these minisatellites are not unstable in the W-V cell line that lacks the Wrn protein or upon down-regulation of Blm expression.

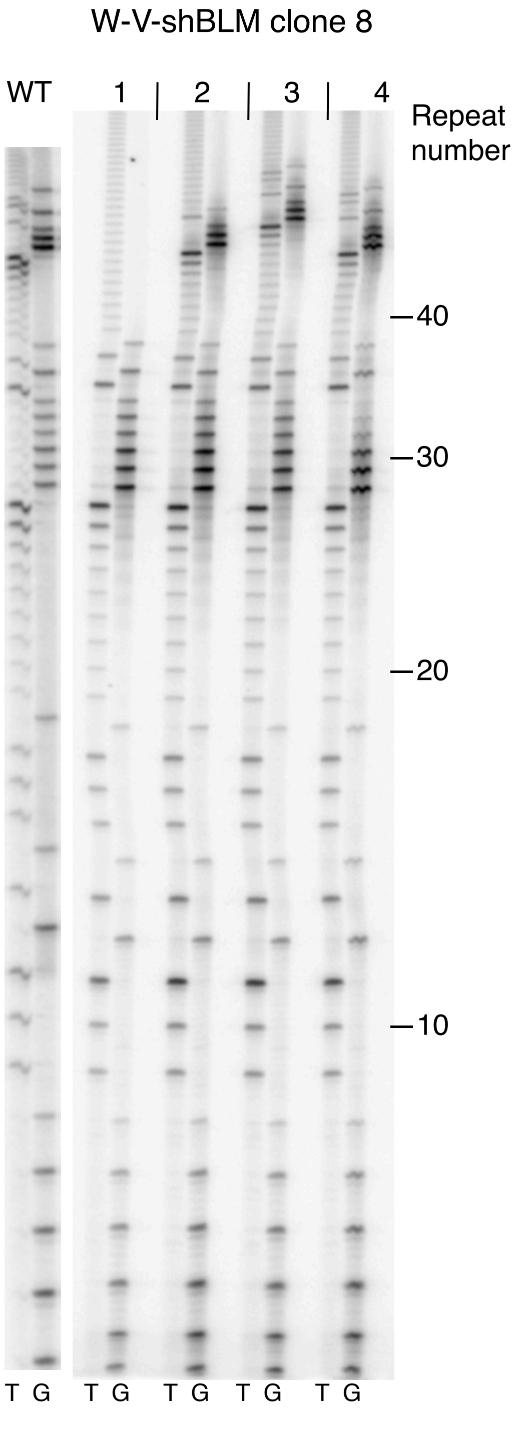

Supplementary figure 4.

Identification of Xp/Yp telomere mutations in the WV-shBLM clone 8.
TVR-PCR is shown for a single STELA molecule containing the progenitor allele (WT) and four mutant molecules. Amplification using an allele specific primer (TS-30T) and a telomere repeat primer that amplifies TTAGGG (T) or TGAGGG (G) repeats are shown. The numbers on the right indicate the telomere repeat position in relation to the start of the telomere.

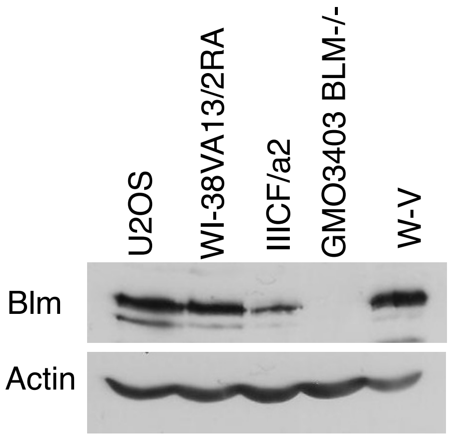

Supplementary figure 5.

Blm expression in ALT+ cell lines.
The expression of Blm is shown for four ALT+ cell lines and the cell line derived from a Bloom syndrome patient (GMO3403) that was used as a negative control. Actin was used as a loading control.
